# Enhancing word recognition skills in English (FL) and Arabic (L1) through transfer effect: an intervention study

**DOI:** 10.3389/fpsyg.2025.1564043

**Published:** 2025-06-18

**Authors:** Noureddine Atouf, Elsayed Issa

**Affiliations:** ^1^Université Chouaib Doukkali, El Jadida, Morocco; ^2^Purdue University, West Lafayette, IN, United States

**Keywords:** word recognition, lexical decision, linear mixed-effects model, accuracy, reading times

## Abstract

**Introduction:**

The extant experimental study measured the effect of a reading training on developing word recognition skills in English (the foreign language, FL) and Arabic (the first language, L1).

**Methods:**

Forty-five participants were selectively allocated to two groups: experimental (n = 25) and control (n = 20). The participants took an online lexical decision task before and after the intervention. The English measures took cognizance of frequency, regularity, and word length, while vowelization marked the Arabic stimuli. To check causality, we fitted four multilevel models to track down the improvement in accuracy and reading times (RTs) based on the interaction of fixed effects (group and time) and random effects (each individual's responses).

**Results:**

The English model's results revealed a statistically significant and positive interaction between the experimental group and post-accuracy rates. Post-reading times, though slightly changed, remained significant compared to the control group. The results of the Arabic models suggested a nuanced difference in the experimental group's performance.

**Discussion:**

The findings revealed compelling insights into the adjustment of processing strategies, namely phonological and orthographic processing skills, to gain lexical access in English and Arabic. The study implicates the import of experimenting with new pedagogical approaches, i.e., reading interventions, to enhance cognitive reading skills among adult learners.

## 1 Introduction

Acquiring the skill to identify word rapidly and correctly is one great step away from reading achievement, but one that is challenging to children and adults as well. Grasping the interplay between L2 reading and L1 processing skills remains a key question in bilingual education and psycholinguistics. Word recognition is an instrumental component of reading fluency, and it involves distinct processing routes depending on the features of the word being read. Studies on whole word recognition (also termed visual word recognition in the literature) unravel the complexity and significance of lexical routes to reading (Rastle, 2016; Coltheart et al., 2001). The dual-route model, for instance, casts the light on the triangular relationship between orthography, phonology, and semantics in word reading, with route selection shaped by word frequency and regularity. In identifying letter strings, readers choose different routes such as orthographic processing, entailing a visually holistic recognition of words (Brysbaert, 2022) and/or phonological processing, involving sub-lexical decoding before activating the recoding of words' meanings (Grabe and Yamashita, 2022; Perfetti and Liu, 2005).

As reading comprehension develops over time, the significance of word-level skills becomes less evident (Braze et al., 2007; Landi, 2010; Perfetti and Hart, 2001). This may suggest that skilled reading is not highly dependent on word recognition processes. However, research on adult reading correlates effective word-level text processes, such as grapho-phonic processes, with reading comprehension, especially among university students with current reading difficulties (McHardy et al., 2021; Nassaji, 2014; Parrila et al., 2007; Stanovich, 2000). Koda (2016)'s model of second language development further supports the importance of word recognition in reading comprehension. L2 word recognition is shaped by readers' L1 experiences. *Transfer* in the context of bilingualism refers to the influence of knowledge or skills from one language (L1 or L2/FL) on the acquisition or use of another. While cross-linguistic transfer has traditionally been examined from L1 to L2, recent studies posit that reverse transfer–from L2/FL to L1–is both theoretically feasible and educationally significant, especially when the target language skills are well-defined and developed (Abu-Rabia and Bluestein-Danon, 2012; Andreou et al., 2019).

The improvement of word reading skills across languages, among all types of learners, should depend, therefore, on similar cognitive and linguistic processing skills (e.g., orthographic processing, phonological awareness, et cetera). These transfer models [e.g. Abu-Rabia and Bluestein-Danon's (2012) Cognitive Retroactive Transfer Hypothesis; Koda's (2007, 2016) Facilitation Model] provide insights into strategies for effective language teaching that support vocabulary acquisition and reading comprehension. The current research study explores the transferability of word recognition skills - defined in this context as the ability to rapidly and accurately identify written stimuli - from the target language to the first language. This is achieved through sufficient print exposure and practice in the foreign language (English), with the ultimate goal of enhancing word recognition in the first language (Arabic), even though the writing and phonological systems differ substantially.

## 2 Literature review

### 2.1 Word recognition and linguistic knowledge

Word recognition is a complex and foundational lower-level processing skill that is naturally triggered by various interconnected subcomponents. These elements comprehend visual word analysis, orthographic processing (letter identification), phonological processing, and phonological recoding which involves activating the semantic representation of the word (Georgiou et al., 2008; Han, 2015; Snowling et al., 2022). In the context of reading, we are frequently referred to as excellent word recognizers. Such a description reflects the essential role word recognition plays in reading (Grabe, 2009; Han, 2015). Furthermore, and according to Perfetti (2007), word recognition is the most frequently “recurrent cognitive activity” in reading (Perfetti, 2007, p. 357). A well-documented line of research on eye movement, for instance, has shown that the visual processing of written input on a word-by-word basis has a positive effect on both word and sentence reading (Han, 2015; Nassaji, 2014; Stanovich, 2000; Warren, 2017).

There are certain factors that determine the ease and preciseness of recognizing words. During offline and online reading, the effect of context triggers the phonological retrieval of lexical items through both syntactic and semantic properties. Vowelized words in Arabic (represented by diacritical marks), for instance, facilitate the processing of words presented in isolation or in contexts (Abu-Rabia, 1996; Aljohani, 2022; Schiff and Saiegh-Haddad, 2018; Taha and Azaizah-Seh, 2017).

The data in (1) shows a visual representation of the processing of the word *signed* /waqqaʕa/ in an incremental sentence processing task (e.g., self-paced online reading):

(1) وقع المدير الوثيقة
waqqaʕa almudiːru alwaθiːqa
signed—the director—the paper
“The director signed the paper”

**Figure 1:**
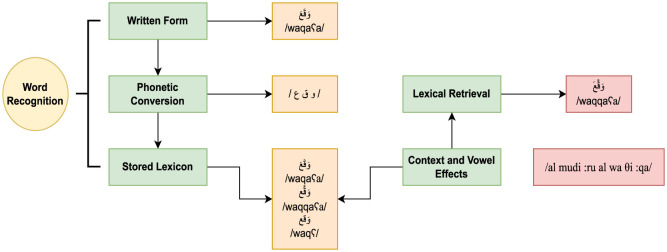
Context and vowelization effect in Arabic word recognition.

**Figure 2:**
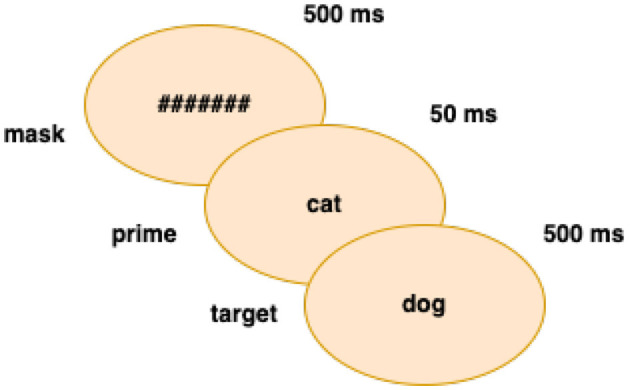
Semantic priming.

Figure 1 shows the conversion of written input into sounds. The combined sounds have various representations in the mental lexicon (i.e., the word is a homograph with different meanings) and are stored based on morpho-phonological information. Only vowelization and context determine which string relates to the initial word form. Similarly, psycholinguistic research on priming measures the effect of neighborhood size on word recognition (Gulan and Valerjev, 2010). When the prime and target word have morphological (for example, imPOSSIBLE/imPERFECT), orthographic, or semantic characteristics in common, the effect is facilitatory (Boudelaa, 2014). This is a priming effect where prior exposure to a related concept (prime) accelerates the processing of the target. As a case in point, if the prime “cat” is followed by the target “dog”—a semantically related animal (PET)— the response time to the target may be faster than when a neutral or unrelated word follows. On the contrary, inhibitory priming occurs when the prime slows down the processing of the target (Gulan and Valerjev, 2010). This happens when the prime and target are not semantically connected. As Figure 2 shows, priming experiments are usually composed of three phases (Brysbaert, 2022): a mask (#######) + a prime + a target. The prime is briefly presented between the mask and the target word.

**Figure 3:**
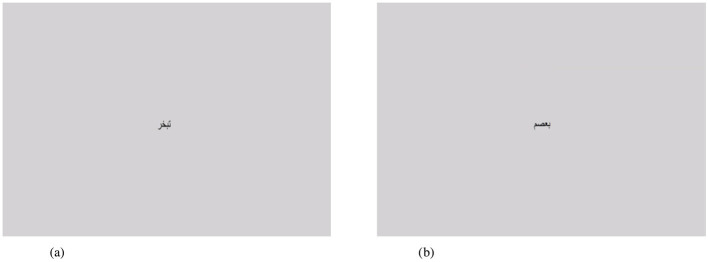
The screen display of words and pseudowords on DmDx. **(a)** Non-vowelized word (+stimulus = 2,000 ms), **(b)** vowelized pseudoword (-stimulus = 2,000 ms)

In contrast to semantic priming, lexical decisions may be simpler in assessing phonological and orthographic effects on word recognition (Nikolaev et al., 2019). For the sake of clarity, we present the display of stimuli items in the current experiment as shown in Figure 3. Pseudowords are used as foils (assigned negative numeric values) to distract participants' attention or to serve as non-words that are phonologically contrasted with real words. Masking and primes are not utilized because pseudowords act as such. Response times are recorded with a timeframe of 2,000 ms (reaction times varied from one experiment to another and usually reflect the purpose of the experimenter).

Frequency effect plays another significant role in word recognition. During lexical access, high-frequency words are easily recognized compared to low-frequency words. Short response times and lower error rates are more related to items with which the participants are familiar (Seidenberg et al., 2022). Additionally, word length and regularity both affect readers' memories. The magnitude of word size is more palpable in rapid naming tasks than lexical decision (Coltheart et al., 2001; New et al., 2006; Vander Stappen and Van Reybroeck, 2018). Regularity refers to the degree of phoneme-grapheme correspondence. The vowel system in English is rife with regular and irregular forms.

By way of illustration, the sound /a/ in monosyllabic words such as *bat, hat, mat, cat* is highly consistent. In contrast, the vowel diagraph “*-ea-*” is predictable in words like *beat, heat, meat, treat*, but not in *threat*. Words consisting of regular spelling patterns are processed faster than words containing irregular forms. A lexical decision trial is likely to consider controlling all these factors. The effects of frequency and regularity (also consistency) are argued to be interrelated (Glushko, 1979; Seidenberg et al., 2022). According to Seidenberg et al. (2022), low-frequency words which follow a regular pattern such as *wave* (notwithstanding the high-frequency neighbor word *have* featured by an irregular form -*ave*) take longer reaction times for skilled college readers. This is also the case when readers are encountered with non-words (*mave* for example). In our attempt to measure college students' ability to identify items, we generated pseudowords in accordance with the dual-route model that emphasizes the import of “generalization” of novel letter strings (Coltheart et al., 2001). In doing so, we controlled for the highlighted factors affecting word recognition except for the context effect, as the lexical decision test involves individual words presented without context. On a comparative scale, we argue that regular words are rule-governed by means of phonological, i.e., sub-lexical, decoding, and this applied to pseudowords as well. Irregular words demand a direct lexical route whereby words are recognized as whole lexical entities (Feder and Abu-Rabia, 2022; Kahn-Horwitz et al., 2012; Seymour et al., 2003). While word recognition skills are characterized by shared cognitive processes, the nature of the orthographic system stands out as a critical factor in determining how these skills are deployed. Bringing the orthographic differences between English and Arabic into focus is crucial to measure the potential for word reading skills transfer between the two languages.

### 2.2 English vs. Arabic orthography

English has a very rich sound system with 40 phonemes in total (Venezky, 1999). English sounds are represented by 26 letters. Five of which are vowels. However, English has approximately fifteen vowel sounds which yield either single or combined sounds (monophtongs as in the “a” sound in cat [æ] or diphtongs as in the “ou” sound in out [a℧]). Vowel combinations or vowel diagraphs are encoded in varied forms. Similarly, consonantal sounds are distinct and superimpose multifarious representations in the English script. The initial sound in the word *photo* corresponds to the sound /f/ suggesting an arbitrary association. The same letter sound /f/ is represented in different words as -f-, -ff, -gh (*full, fluff* , *laugh* accordingly), making English an opaque orthography. The inconsistencies adumbrated above affect English word recognition, since readers in the English language must work out the mapping detail between graphemes and their phonemic counterparts.

Arabic sounds are represented by 28 letters, two of which act as semivocals. Specifically, these are the glides /w/ and /j/ that can open syllables (when they are semi-consonants) or prolongate corresponding vowels /uː/ in the word /ðahabuː/ (*they went*) and /iː/ in the /tiːn/ (*fig*) acting thus as semi-vowels (Holes, 2004). The Arabic grapheme *wa* corresponds to the glide /w/ and long vowel /uː/. Likewise, *ya* can relate to the consonantal glide /j/ or the long vowel /iː/ in written words (Saiegh-Haddad and Henkin-Roitfarb, 2014).

Unlike English, which is rife with vowel sounds, Arabic is featured by a derivational consonantal inventory (i.e., words are derived from a tri-consonantal root) with a limited number of vowels. There are three short vowels represented by diacritics placed above or beneath the letter (low /a/ fatḍa, high front /i/ kasra, and high back /u/ ḥamma). Short vowels are salient phonological features in Arabic as they indicate case-marking (e.g., nominative, accusative cases, etc.). Long vowels are the corresponding elongated sounds that have graphic forms in the orthography (Broselow, 2008).

The level of grapheme-phoneme consistency in Arabic is relatively high in comparison to the sound-letter relationship in the English writing system. Arabic readers rely heavily on phonological information to activate the semantic properties of words (Fender, 2008). However, lexical access in Arabic is as complex as the language's morphology (Saiegh-Haddad and Geva, 2008). Arabic words are argued to spring from a consonantal root (e.g. “KTB” to write) and a vocalic pattern (a vowel sound a “KaTaB” wrote) to build different forms [Abu-Rabia (2001); for a full review, see Boudelaa (2014)]. The consonantal root, which conveys semantic information, is not encoded in the orthography. Therefore, the derivational process triggered by morpho-phonological mapping is essential in both spoken and visual word recognition. Given these structural discrepancies, it is important to examine how adult learners interact with word recognition while learning a second and/or foreign language. The ensuing section reviews research on adult L2 readers.

### 2.3 Research on adult L2 word recognition

Few studies examine adult (L2) word recognition, measuring the relationship between word-level processes and reading comprehension (Fender, 2003; Shiotsu, 2009). Psycholinguistic research shows that English language learners (ELLs) who exhibit automatic and proficient word reading skills tend to achieve high scores in comprehension tests (Nassaji, 2014). This is an indicator that lower-level processes shape reading abilities (Nassaji and Geva, 1999). Shiotsu (2009) explored the significance of L2 word recognition skills in an experiment involving EFL Japanese university students. The results showed that the students' noticeable performance in understanding L2 texts was ascribed to adequate word decoding skills. Similar findings were observed in Fender (2003)'s study, which evaluated L2 word reading proficiency among two groups of Arabic and Japanese ESL learners. Parrila et al. (2007)'s study on persistent reading issues among adult students revealed that comprehending a text at a level expected for university students was challenged by current problems in L2 word decoding and phonological processing. Atouf and Harrizi (2022) ran an online experiment intended to substantiate English (L2) word recognition skills among adult EFL learners. Their findings implicated that L2 reading instruction enhanced decoding abilities. Findings from adult L2 reading research proffer a foundation for exploring cross-linguistic transfer. Specifically, they raise the question of whether improvements in L2 word reading can wield an impact on equivalent L1 processing skills.

### 2.4 Transfer effect

There is no such a consensus in the literature as to what genuinely constitute transfer (Koda, 2007). By and large, transfer can be defined as the process of facilitating the acquisition of L2 through the use of linguistic and cognitive skills acquired in L1 (Genesee and Geva, 2006; Odlin, 1989). Transferring these skills across orthographies pivots around the linguistic distance between L1 and L2. The closer the languages are, the more flexible is the use of language components in both directions (from L1 to L2 and vice versa). In alphabetic orthographies like English and Arabic, as a case in point, similar processing demands are posed on readers to decode written input. That is, both languages allow a grapho-phonological encoding of spoken forms. The Phonological Principle, i.e. all readers must learn to map phonemes onto letters, is considered to be ipso fact universal across all languages (Da Fontoura and Siegel, 1995; Genesee and Geva, 2006). This universal linkage prerequisite is also critical in word reading accuracy (Seidenberg and McClelland, 1989). By contrast, transfer does not occur when the L1 and L2 involve language-specific processing mechanisms (Pasquarella et al., 2015).

Recent models in the literature accounts for transfer effects of linguistic and cognitive features across languages. Koda (2007)'s *Facilitation Model* highlights the shared L1 and L2 processing strategies in L2 word recognition. The model postulates that the degree of orthographic similarity between L1 and L2 influences L2 word processing efficiency. That is, when strong links are formed between orthographic forms and semantic elements, as seen in high frequency words and sight words, quicker and effective word identification occurs (Koda, 2007). However, the model does not overtly claim common processing strategies across distant languages.

The *Cognitive Retroactive Transfer Hypothesis (CRT)* builds upon and extends Koda's model (Abu-Rabia and Bluestein-Danon, 2012; Abu-Rabia and Shakkour, 2014). Within an intervention framework, the CRT hypothesis proposes a new dimension for reading skills' transfer from the target language (L2/FL) to the first language irrespective of linguistic disparities (Abu-Rabia and Shakkour, 2014; Abu-Rabia and Wattad, 2022). Drawing upon Cummins (1991)'s *Interdependence Hypothesis*, which suggests that skilled readers can make use of their first language background to facilitate and expedite the learning of other languages, the CRT claims that key linguistic predictors of reading are universal and, hence, transferable across typologically different orthographies (Abu-Rabia et al., 2013; Abu-Rabia and Wattad, 2022). From a critique standpoint, lower-level processing skills inherent in online word reading have not been subject to investigation by the CRT. The extant experiment attempts to fill this gap.

Empirical research gives credit to print exposure and practice in developing word reading skills such as accuracy and speed among readers of distinct reading abilities, such as poor, dyslexic, normal readers (Altmisdort, 2016; Andreou et al., 2019). For instance, Feder and Abu-Rabia (2022) acknowledged the import of L2 interventions which significantly brought about conspicuous reading improvement, across multiple linguistic and cognitive skills including word recognition, not only in the target language (English) but also in the learners' first language (Hebrew).

Furthermore, research on cross-language transfer of word reading considered script similarity between languages for transfer effect to occur (Keung and Ho, 2009). Pasquarella et al. (2015) conducted a study on cross-linguistic transfer of fluency and accuracy among Spanish-English and Chinese-English bilinguals. Accuracy was found to be transferable only among the Spanish-English bilingual group, while cross-language transfer of word reading fluency was highly significant for both groups. On the one hand the transfer of accuracy was predicated upon structural similarities between L1 and L2. On the other hand, fluency was transferable as it operated at a more script-universal level (Pasquarella et al., 2015). The body of research outlined here postulates that the transfer process is not unidirectional. That is, skills acquired in an L2, particularly those rooted in cognitive processes pertaining to word identification, can potentially influence L1 reading behaviors. However, evidence for such reverse transfer, involving different orthographies, remains underexplored.

While much of the research on cross-linguistic transfer deals with the effect of L1 linguistic knowledge on L2 reading development, emerging research suggests a dynamic and bidirectional relationship between L1 and L2. Koda's (2016) Facilitation Model posits that L2 word recognition is shaped by L1 processing when the two languages share the same orthographic characteristics. This model leaves open the question of whether enhanced L2 lexical access strategies can in turn facilitate L1 reading. Similarly, existing CRT frameworks often ignore the transferability of cognitive processes (i.e., orthographic and phonological processing skills) in word recognition. This study challenges that assumption by experimenting with a new mode of transfer: whether structured English (L2) word recognition intervention (i.e., reinforced word reading instruction) can improve word identification in the target language (English, FL) and, by reverse effect, word reading in the first language (Arabic, L1). The contribution of the current research is its empirical testing of reverse transfer, a relatively understudied area in cross-language transfer of reading skills. By utilizing multilevel modeling to measure accuracy and reaction times for both languages, the present paper not only provides evidence for the hypothesis of common cognitive processing routes but also paves the way for posing theoretical questions about how different languages with varying orthographic depths interact through transfer.

### 2.5 Present study

The present study builds on this line of studies (previously underscored in the review) by investigating whether specialized training in English word recognition can yield measurable improvements in Arabic word reading among university students. We aim to examine the effect of a reading training (in English) on improving foreign language word recognition skills among adult learners with unidentical proficiency levels (Abu-Rabia and Shakkour, 2014; Abu-Rabia and Wattad, 2022; Altmisdort, 2016; Andreou and Segklia, 2019). We also seek to measure the transferability of cognitive strategies, namely phonological and orthographic processing, following sufficient print experience and practice from English (FL) to Arabic (L1). Experimental studies on transfer effects have assessed the linguistic similarities between the languages under scrutiny and how word recognition skills are facilitated by L1 and L2 structural closeness (e.g. Pasquarella et al., 2015). We claim that the new mode of transfer may be bidirectional (i.e. from L2/FL to L1) and the transfer effects may be attested at the level of cognitive strategies pertaining to reading accuracy and speed. To this end, we design a treatment condition (reading program) which extends over two phases where the participants (in two main groups) take a simple online lexical decision task in English (FL) and Arabic (L1).

The ongoing experiment is set to answer the following questions:

- To what extent would providing adequate FL (or L2) print exposure and practice develop English word recognition in terms of accuracy and speed?
- What routes would participants in both groups use to recognize words in English and Arabic?
- Would the FL (or L2) reading training exert a varying effect on the same word recognition skills in Arabic?

## 3 Methods

### 3.1 Participants

Forty-five university freshmen recruited from the English Department at (Hassan II University of Casablanca) participated in this study. The participants were enrolled in the Spring term in which they had to take a reading comprehension course. Before the commencement of the reading intervention, the students were asked to complete a background questionnaire which collected various factors including current and past literacy habits at school and at home. All participants had the same educational background and used Modern Standard Arabic (MSA) in their education and literacy practices, while Moroccan Darija remains their daily spoken language.^1^ They also had pre-intermediate and elementary levels of English proficiency and a varied level of Arabic. In English, for instance, a small portion of the population self-claimed to be proficient and poor (2.2% and 6.7% accordingly). As opposed to English, 28.9% self-reported that their Arabic was poor. Inclusion criteria required that only students who felt they were still struggling with English Reading Comprehension Course could take part in the experiment. Exclusion criteria included students who refused to be committed to the intervention program and students who had above average in the pretest scores. Table 1 displays the gender distribution where both male and female students were included with an approximate gender balance. The age range of most participants (64.4 %) was between 17 (being the lowest range) and 20 years old; the rest of participants were aged 21 years or older.

**Table 1:** Gender distribution in the study

**Gender**	**Frequency**	**Percentage**
Female	23	51.10%
Male	22	48.90%

### 3.2 Measures

#### 3.2.1 English word recognition

To measure English word recognition, we controlled for word length, frequency, and regularity. We utilized the Word Frequency List of American English (Davies and Gardner, 2010). The entries in this list are arranged according to their order of frequency. The test comprised 60 frequent words split into two major groups. English real words followed predictable as well as unpredictable spelling patterns (see Supplementary Table S1). Likewise, English non-words followed consistent and inconsistent spelling patterns (examples of unlisted items are: PHINT/shinte; *ZOW). To maintain the same criteria of word selection, we either excerpted the pseudowords from *The Source: A Curriculum for Reading Mentors* (Florida Department of Education, 2003) or were generated by the experimenters themselves. The construction of the pseudowords violated the phonological system of the English language (e.g. restricted consonantal clusters). Regarding word length, the items consisted of either one-syllable or two-syllable pronounceable words. The participants were given instructions to make lexical decisions rapidly and accurately. The evaluation of the participants' ability to process phonological forms with their varied corresponding orthographic elements was made possible by incorporating both regular and irregular patterns in the online test. Responding correctly on presented stimuli before and after the intervention was a signpost for the students' knowledge of English spelling regularities.

#### 3.2.2 Arabic word recognition

Akin to the previous test, Arabic word recognition task contained 60 words divided into two categories: pseudowords and real words. Vocalization was taken into cognizance to meet the criterion of orthographic regularities. The words were vocalized by means of diacritics featured by the Arabic short vowels (/u/ ḥamma, /i/ kasra, /a/ fatḍa) (Holes, 2004; Saiegh-Haddad and Henkin-Roitfarb, 2014). On the one hand, nonsense words were vowelized, enabling one single reading of the relevant stimulus. Additionally, pseudoword foils involved transposition (i.e., a process entailing misplacing letters in a given word irtælæḥæ). Transposed items were taken from Boudelaa et al. (2019). Table 2 shows examples from the listed Arabic stimuli (see the Supplementary Table S2 for the complete list).

**Table 2:** Examples of Arabic real words and pseudowords.

**Arabic real words irregular forms (unvowelized)**	**Arabic pseudowords regular forms (vowelized)**
The word ظَهر /ð*inline*2.*eps*ahr/ could be read as a verb meaning “appear” ظَهَر /ð*inline*2.*eps*ahar/, a defined noun meaning the “back” الظَّهْرُ /ʔað*inline*2.*eps*ð*inline*2.*eps*ahru/, the “afternoon” الظُّهْرُ /ʔað*inline*2.*eps*ð*inline*2.*eps*uhru/, the “house furniture” الظَّهَرُ /ʔað*inline*2.*eps*ð*inline*2.*eps*aharu/ when not vowelized.	The word اِسْتَعَم /ʔistaʕam/ a pseudoword involving transposing letter m and the glottal stop (with a an original meaning: ‘he listened'). The word تَبَشَّم /tabaʃʃam/ a pseudoword created according to the verbal pattern V: tC1C2C2C3

We used the phonetic symbols for Arabic according to International Phonetic Alphabet.

On the other hand, real words were not vowelized, allowing, thus, for different readings. Words that had only one reading form [e.g., sæmaː/ʔ (sky)] were excluded. As to word frequency and length, all words were made up of seven to eight letters long, denoting different grammatical forms (verbal and nominal forms conforming to the Arabic patterns known as /ʔawzaːn/). In the lexical decision task, contextual and syntactic information were purposefully unprovided since the items were not indicative of any reference to such pragmatic clues. The emphasis was placed on both speed and accuracy in reading the items. The Arabic items were generated by Al-Maani Online Dictionary and the authors as well.

### 3.3 Procedure

Each participant took the online lexical decision task individually in a quiet room at the English Department in the School of Letters and Humanities. The administration occurred in a varied sequence and spanned two days consecutively. On the first day, the participants completed the English test. Then, on the next day, the corresponding Arabic test was delivered. During this phase, three computers were employed to expedite the test. Prior to commencing the test, the experimenter provided verbal and written instructions in Arabic to ensure comprehension.

The DMDX program software (Forster and Forster, 2003) was utilized to control the online word identification task, with lexical items displayed on the computer screen. The type of items (whether the stimuli were real or nonsense words) was presented in a random sequence to adhere to the test validity. The participants were guided to respond using two designated buttons on the computer keyboard. In the case of real words, the participants made correct answers by rapidly pressing the right shift key (yes-answers). Conversely, for non-words, correct responses were recorded by a swift pressing of the left shift key (no-answers). The subjects were given 2 seconds (2,000 MS) to make their decisions before the next stimulus appeared on the screen. Responses classified as “time-out answers,” that is not deciding whether a string of letters is a pseudoword or existing word within the provided time frame, occurred when the 2-second limit elapsed. By and large, the test extended for a duration of three to five minutes in case technical issues arose.

Upon completion, DMDX requested the participants to save the data. In the post-lexical decision task, the same number of our sample (45 students) was considered. In this stage, we maintained the same sequence, with each participant initially taking the English word recognition before sitting for the matched Arabic word recognition test. To ensure consistency and understanding, the same task structure and interface were used throughout all phases. In doing so, no verbal or written instructions were given during this phase. We assumed that the students were already acquainted with the lexical decision task, because it basically involved the same procedure of testing described earlier. It is noteworthy to mention that the control group did not receive any targeted training in English word recognition and went on with their regular university coursework that was irrelevant to the experimental activities during the intervention.

### 3.4 Word reading intervention

The experiment aimed at improving the participants' word level sub-skills such as phonological awareness and spelling skills through designing a word reading intervention. The remedial lessons were made up of three main tiers: English sound system (consonants and vowels), spelling rules (syllables), and word building skills (affixation). The participants were taught major characteristics of the English sound system. The focus was put on the representations of English phonemes as they appear in the International Phonetic Alphabetic (IPA) chart. This included teaching common combinations of letters such as consonantal clusters and vowel diagraphs (e.g., -ee-, -ea-, ou-, -oo-, etc). Additionally, the students were introduced to a variety of rhyming patterns' tasks and word families' activities (phonograms: _ack, _ess, _ick, _ock, etc). As to spelling skills, lessons such as vowel trivia, triangular words, and compound words were adopted from Teaching English spelling: A practical guide (Shemesh and Waller, 2000). These lessons were keyed to fit the level of the students. A vowel trivia task, for instance, presented the participants with a definition and a spelling frame (e.g., a serviette: n_pk_n, see Supplementary Table S3). They needed to insert a letter (vowels or consonants to provide the correct spelling of the word). Compounding required the participants to choose words from two boxes to make up one word. When combined together, these lexical items were basically pseudowords representing existing English compounds. The task was to replace the made-up compounds with their real counterparts. Afterwards, the participants played “sight word bingo” and a “PowerPoint pandemonium” games (Watkins, 1918). To bolster up word building skills, the intervention provided the participants with tailored lessons on syllabification and affixations. First, the participants learnt the six types of syllables (open syllables, close syllables, syllables with silent -e, vowel team syllables -ow, -ea, r-controlled _ar and _or as in sword, and syllables with a word-final consonant+ le like “ankle”). Syllabification activities included common ESL activities such as “Speed Drill”. In this drill, students read two sets of words–one set focused on specific syllable-spelling patterns, and the other set contained 20 common syllables arranged randomly. The participants were then asked to read both sets as quickly as possible, while simultaneously matching the words to their corresponding syllable patterns (e.g., matching C-le with “middle") and marking these pairs with a special symbol like a triangle, asterisk, or circle.

### 3.5 Data analysis

To address the research question, we constructed 4 linear mixed-effects models (LMMs) from lme4 package (Bates et al., 2014) for R to test for mean differences between the experimental and control groups. We fitted two LLMs that predict the accuracy scores (1 = correct, 0 = incorrect) for English and Arabic and two LLMs to predict reading speed using response times (RTs) for English and Arabic as well. The fitted four model follow the following syntax: variable = (β0+β0_*j*_)+β1*group*:*time*+ϵ where the variable is the metric score, group is for the categorical factor (group) with two levels (experimental and control) and *group*:*time* is the interaction between group and time as a categorical factor with two levels (pre-test and post-test) as well. The ϵ is the random effect that the subject indicates as we look at multiple measures per subject. To analyze English and Arabic tests, we broke the general linear model into several related models to check for the groups' differences before the intervention (to confirm the characteristics of our population) and after the intervention (to check for significance in the groups' performance). The linear models in the Arabic test were accompanied by independent samples t-tests for each phase to measure the Group × Time interaction and to check for possible improvement in the post-test, implicitly suggesting a cross-language transfer of cognitive processes.

## 4 Results

### 4.1 Descriptive statistics

**Figure 4:**
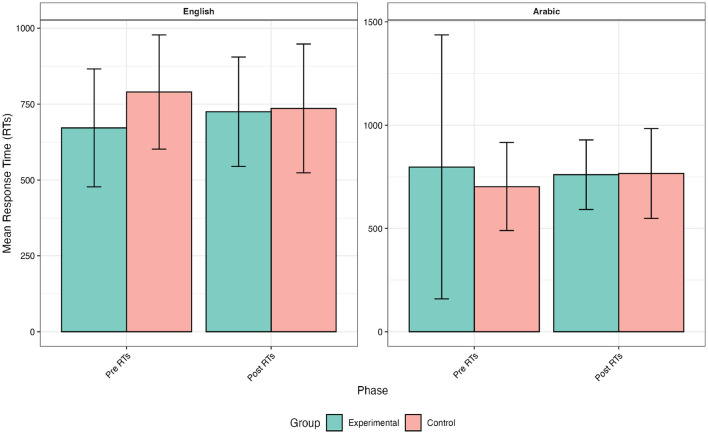
Means of RTs before and after the intervention.

Figure 4 describes the means in the control and experimental groups' response times at two-time points. The mean RTs were *M* = 790 (*SD* = 188) before the intervention for participants in the control group. This mean value decreased to *M* = 736 (*SD* = 212). In contrast, the reading times for the experimental group were slower (672 milliseconds) before the intervention with a conspicuous increase in post_RTs (*M* = 725, *SD* = 180). In Arabic, the participants had varying degrees of response times on the lexical items, but the range did not exceed 700 milliseconds: control group, before *M* = 703 (*SD* = 214) and after *M* = 767 (*SD* = 127); the experimental group, before *M* = 798 (*SD* = 639) and after *M* = 761 (*SD* = 168). The experimental group's reading times were a bit slower while the reading times in the control group jumped during the post-intervention stage.

**Figure 5:**
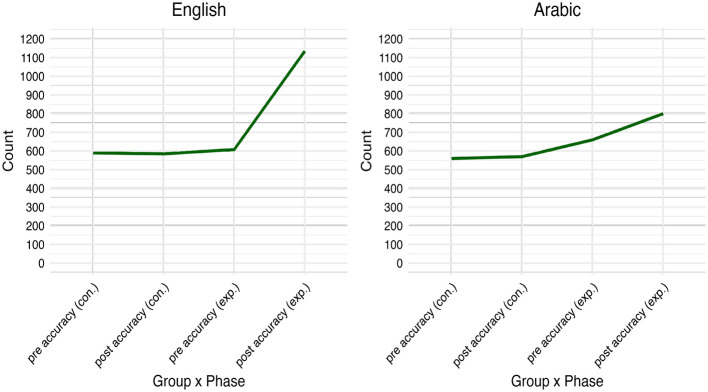
Mean values of accuracy before and after the intervention.

Figure 5 displays the variations in the answers made by the control and experimental groups before (Pre_Accuracy) and after (Post_Accuracy) the intervention in English word recognition. The control group had a total of (*n* = 581) correct answers, while the experimental group exhibited a slightly higher count of (*n* = 607) correct answers. After the intervention, the control group's post-count slipped to (*n* = 572) correct answers, while posttest data showed a notable increase to (*n* = 1135) correct answers among the experimental group. Additionally, Figure 5 presents the counts of correct answers made by the Control and experimental groups before (pre_accuracy) and after (post_accuracy) the intervention on Arabic LD task. The control group made a total of (*n* = 544) correct answers, while the experimental group exhibited a relatively higher count of (*n* = 642) correct answers. After the intervention, the control group's post-count rose slightly to (*n* = 547) correct answers, whereas the experimental group displayed a substantial increase to (*n* = 793) correct answers. Compared to English, the experiment group, although having higher post-accuracy rates, did not have a similar count for correct trials on the Arabic word identification test. Table 3 presents an in-depth description of (RTs) in the English word recognition test for the control and experimental groups, categorized by word type (pseudowords and real words) before (Pre_RTs) and after (Post_RTs) the intervention.

**Table 3:** Word category RTs before and after the intervention for both groups (numbers are in milliseconds).

**Language**	**Group**	**Word category**	**Phase**	**Mean**	**SD**
English	Control	Pseudowords	Pre_RTs	825	187
			Post_RTs	785	218
		Real words	Pre_RTs	768	185
			Post_RTs	706	203
	Experimental	Pseudowords	Pre_RTs	703	200
			Post_RTs	769	180
		Real words	Pre_RTs	651	188
			Post_RTs	686	170
Arabic	Control	Pseudowords	Pre_RTs	734	225
			Post_RTs	791	227
		Real words	Pre_RTs	666	194
			Post_RTs	745	206
	Experimental	Pseudowords	Pre_RTs	801	236
			Post_RTs	789	170
		Real words	Pre_RTs	795	842
			Post_RTs	737	162

The response time (RT) data revealed distinct patterns between control and experimental groups across both languages. In English, the control group showed decreased RTs for both pseudowords (e.g. from 825. ms to 785. ms) and real words following the intervention. Conversely, the experimental group demonstrated increased RTs. As for Arabic, the control group took more time to recognize both word categories during the post-intervention phase. The experimental group displayed varied identification attitudes, with nuanced decreases in RTs for pseudowords and more substantial decreases for real words. These contrasting patterns suggest that the intervention had language-specific effects on word processing efficiency, with potentially more beneficial outcomes for the experimental group in Arabic.

### 4.2 Regression analysis

Tables 4, 5 show the regression analysis for the English and Arabic word recognition accuracy respectively.

**Table 4:** A linear mixed model with group and time as fixed effects and the English accuracy scores for reading times as the dependent variable.

**Fixed effects**				**Random effects**		
	**Est**.	**Test (** ***df*** **)**	***p***		**Variance**	**SD**
Intercept_ref = Control_	5.15	t = 27.35 (8.02)	< 0.001	Subject	0.0032	0.0573
Group_Experimental_	-2.84	t = -11.2 (8.02)	< 0.001			
Time_Pre_Accuracy_	-7.62	t = -0.39 (5.26)	0.696			
Group × Time	3.65	t = 13.96 (5.26)	< 0.001			

Model: score group*time+(1|*subject*).

**Table 5:** A linear mixed model with group and time as fixed effects and the Arabic accuracy scores for reading times as the dependent variable.

**Fixed effects**				**Random effects**		
	**Est**.	**Test (** ***df*** **)**	***p***		**Variance**	**SD**
Intercept_ref = Control_	5.28	t = 21.4 (6.24)	< 0.001	Subject	0.0080	0.0895
Group_Experimental_	-7.53	t = -2.27 (6.24)	0.002			
Time_Pre_Accuracy_	2.58	t = 0.12 (5.17)	0.898			
Group × Time	1.01	t = 3.7 (5.17)	< 0.001			

Model: score group*time+(1|*subject*).

For English word recognition, the estimated subject variance was 0.0032 while the estimated residual variance was 0.224, indicating a substantial amount of between-subjects variability. We found a main effect for group (β = -2.84, *p* = 0.001, *d* = -6.04) and a significant main effect for the interaction term Group × Time (β = 3.65, *p* = 0.0002, *d* = 7.76). The effect of *Group* × *Time* is statistically significant and positive (β = 0.37, 95% CI [0.31, 0.42], t(5304) = 13.97, *p* < 0.001; Std. β = 0.73, 95% CI [0.63, 0.84]). These results entail that learners who received English reading training or print exposure started off with lower word recognition accuracy but showed improvement over time.

Similarly, for Arabic word recognition, the estimated subject variance was 0.0080 while the estimated residual variance was 0.240, indicating a substantial amount of between-subjects variability. We found a main effect for group (β = -7.53, *p* = 0.026, *d* = -15.36) and a significant main effect for the interaction term *Group* × *Time* (β = 1.016, *p* = 0.0002, *d* = 2.07). The effect of Group [EXPER] × Time [PRE_Accuracy] is statistically significant and negative (β = -483.47, 95% CI [-566.76, -400.17], t(5304) = -11.38, *p* < 0.001; Std. β = -0.60, 95% CI [-0.71, -0.50]). The results show that while the intervention had no positive effect on English word recognition, it could probably interfere with Arabic word reading skills. This can be ascribed to the disparities at the level of orthography underlying the two languages. To navigate through word reading in English and Arabic requires distinct processing demands.

For *word speed reading*, Tables 6, 7 show the regression analysis for the English and Arabic word recognition accuracy respectively.

**Table 6:** A linear mixed model with group and time as fixed effects and the English response times (RTs) as the dependent variable.

**Fixed effects**				**Random effects**		
	**Est**.	**Test (** ***df*** **)**	***p***		**Variance**	**SD**
Intercept_ref = Control_	-59.6	t = -1.83 (73.9)	0.07	Subject	11,039	105.1
Group_Experimental_	418.1	t = 9.60 (73.9)	< 0.001			
Time_Pre_RT_	3.46	t = 0.10 (5,263)	0.09			
Group × Time	-483.4	t = -11.3 (5,263)	< 0.001			

Model: times group*time+(1|*subject*).

**Table 7:** A linear mixed model with group and time as fixed effects and the Arabic response times (RTs) as the dependent variable.

**Fixed effects**				**Random effects**		
	**Est**.	**Test (** ***df*** **)**	***p***		**Variance**	**SD**
Intercept_ref = Control_	-101.8	t = -2.3 (63.39)	0.02	Subject	24,708	157.2
Group_Experimental_	155.6	t = 2.6 (63.39)	0.008			
Time_Pre_RT_	-39.4	t = -1.0 (5,173)	0.27			
Group × Time	-179.1	t = -3.6 (5,173)	< 0.001			

Model: times group*time+(1|*subject*).

For English word recognition, the estimated subject variance was 11,039 while the estimated residual variance was 591,690, indicating a substantial amount of between-subjects variability. We found a main effect for group (β = 418.1, *p* = 0.001, *d* = 0.54) and a significant main effect for the interaction term Group x Time (β = -483.46, *p* = 0.001, *d* = -0.62). The effect of Group [EXPER] × Time [PRE_Accuracy] is statistically significant and positive (β = 0.10, 95% CI [0.05, 0.16], t_(_5214) = 3.72, *p* < 0.001; Std. β = 0.20,95% CI [0.10, 0.31]). These results are indicative of the slower reading rates featuring the experimental group pre-intervention before improving afterwards (i.e., post-intervention). The development of word reading speed can be attributed to sufficient print exposure and practice.

Similarly, for Arabic word recognition, the estimated subject variance was 0.0080 while the estimated residual variance was 0.240, indicating a substantial amount of between-subjects variability. We found a main effect for group (β= 155.6, *p*= 0.008, *d* = 0.17) and a significant main effect for the interaction term Group × Time (β= -179.1, *p* = 0.001, *d* = -0.20). The effect of Group [EXPER] × Time [PRE_RT] is statistically significant and negative (β = -179.16, 95% CI [-275.75, -82.57], t (5214) = -3.64, *p* < 0.001; Std. β = -0.20, 95% CI [-0.31, -0.09]). Although there was some improvement in Arabic response times among the experimental group, the effect was weaker than in English. This suggests that reading training in English had limited transfer to Arabic word recognition speed. The skills gained may be language-specific and not language-universal.

For the sake of understanding the nuances in the effectiveness of the intervention on developing cross-language word identification skills, virtually Arabic, we further performed an independent samples t-test based on post-test scores to provide valuable statistics on Arabic word recognition processes after the intervention.

Based on the results, as indicated in Table 8, of the two-sample t-test, we found a significant difference in the mean scores between the control group (*M* = 0.528) and the experimental group (*M* = 0.453) after the intervention, *t*(2608) = 3.836, *p* = 0.000128. The t-value indicates that the observed difference in means is statistically significant. The 95% confidence interval for the mean difference ranged from 0.037 to 0.114.

**Table 8:** Independent samples t-test of Arabic word recognition for both groups after the intervention.

**Group**	**Mean**	**t-value**	**df**	**Two-tailed**	**95% Confidence interval**	
Control	0.528				Lower	Upper
		3.8361	2,608	*p* = 0.000128		
Experimental	0.453				0.0368	0.1138

## 5 Discussion

This study checked for a potential improvement of word reading skills in the target language (English, the FL) by providing sufficient FL print experience to an experimental group of adult language learners. The current psycholinguistic experiment aimed at unraveling which processing strategies, related to word recognition, are employed by the participants to identify lexical items. To this end, we administered an online lexical decision over two phases (i.e., pre-intervention and post-intervention). The focus was on the two components of word recognition, namely accuracy and speed. By reacting to presented stimuli (real words and non-words) within a given timeframe (2,000 ms), the lexical decision not only indicated accuracy and speed rates but also provided insights into the lower-level processing skills involved in word reading. Finally, lexical access was compared in two typologically distinct languages, English and Arabic, but ones that universally impose similar encoding demands, i.e., both English are Arabic are alphabetic. The purpose of incorporating Arabic in the linguistic background of the experiment was to measure transferring effects.

### 5.1 Accuracy and speed

The Descriptive results indicate an improvement in the experimental group's accuracy and speed rates after receiving the reading training. English correct trials were very significant after developing English spelling and orthographic skills. Both word categories in English were not recognized faster for the control group before and after the intervention. Given the regularity variable in English words (consisting of irregular forms), participants in the experimental group took longer times to identify presented stimulus. This result is in congruence with the Orthographic Depth Hypothesis which suggests that irregular forms affect response times (Seidenberg et al., 2022). Moreover, the increase in reading times within the experimental groups suggests a shift to phonological processing instead of direct visual word recognition. In Arabic, the decrease of response times attested at the level of pseudowords implied the role of vowelization in facilitating word identification among the experimental group (Abu-Rabia, 1996; Aljohani, 2022; Schiff and Saiegh-Haddad, 2018; Taha and Azaizah-Seh, 2017). The study's results with respect to response times contradict (Fender, 2008). There is a conspicuous adjustment of processing strategies in Arabic, because word recognition did not operate by means of phonological processing (which is typically used by Arabic readers). We cannot claim that lexical access in Arabic was gained by holistic orthographic processing given the nuanced changes in reading rates. We can, however, claim that processing skills are universal in their nature and can be significantly adjusted in such alphabetic languages as English and Arabic (Da Fontoura and Siegel, 1995; Genesee and Geva, 2006; Pasquarella et al., 2015).

The results of linear mixed-effects models performed in both languages indicated that the experimental group had a higher accuracy rate in the English word recognition task after the intervention (effect size: Cohen's d = 7.76). Likewise, reading times, though increased over time, remained statistically significant compared to the control group (effect size: Cohen's d = -0.62). The effect size numbers are large and the number refers to a negative magnitude meaning that the mean of the reference level group (control) is smaller than the mean of the other group (experimental) according to Cohen (2013)'s interpretation of effect sizes which are small (d = 0.20), medium (d = 0.50), and large (d = 0.80). The values “positive” and “negative” attached to statistical significance in the interaction Group x Time indicate either equal or varying degrees of difference. For example, in English words, when the response times increased for the experimental group, they decreased for the control group indicating “negative” values. When values of accuracy and reading times are equally increasing or decreasing for both groups statistical significance indicates “positive” values. The linear mixed-effects models in Arabic revealed a slight improvement in reading times which were relatively slower than the control group. Accuracy rates were also significantly higher for the experimental group. These results suggest that the intervention had a positive effect on the experimental group's scores, as they outmaneuvered the control group after the intervention. However, and compared to English, Arabic word reading accuracy was not strong enough as shown in Figure 5. The accuracy rates of Arabic, though statistically significant, were likely to spring from the binary orientation of the lexical decision where random correct answers may interfere. Word reading accuracy is corollary of adequate print exposure within the specific domains of the target orthography. English and Arabic orthographies have different writing systems, which involve distinct script-specific encoding processes. The nuanced change in accuracy scores across the languages aligns with other similar findings in the literature (e.g., Pasquarella et al., 2015). In addition, the importance of reading instruction in developing word accuracy is reflected in the present study's findings (Atouf and Harrizi, 2022).

The slight increase in reading times among the experimental group, coupled with improved accuracy, can be ascribed to a strategic shift toward phonological processing. According to Perfetti (2007)'s hypothesis of Lexical Quality, slower but more accurate word recognition refers to the engagement of more effortful, sub-lexical processing, notably during the early stages of learning to read. Likewise, Nassaji (2014) argues that increased response times can inform the development of complex decoding strategies in L2 learners. In this study, the increase in RTs may suggest that participants moved away from shallow visual identification strategies toward a more systematic, overt, and phonologically grounded approach to word recognition.

### 5.2 Transfer effects

The differential improvement in word recognition skills following the intervention is shown in the confidence interval. The 95% confidence interval for English is [0.63, 0.84]. This means that the true population parameter for the English group lies between 0.63 and 0.84. The 95% confidence interval for Arabic is [0.10, 0.31]. This means that the true population parameter for the Arabic group lies between 0.10 and 0.31. Assuming that larger values indicate better outcomes, we conclude that English word recognition accuracy seems to outweigh its corresponding Arabic skill. With this being considered, the findings of the current study suggested a slight improvement in Arabic accuracy scores (the p value was statistically significant), giving credits to recent transfer models such as the Cognitive Retroactive Transfer Hypothesis (Feder and Abu-Rabia, 2022; Abu-Rabia and Shakkour, 2014; Andreou et al., 2019; Altmisdort, 2016).

The study offers an intriguing mode of transfer taking place in both ways from L1 to FL and vice versa. The increase in English word recognition times revealed a reliance of L1 processing strategy based on phonology. To navigate through the irregularities featuring English spelling, the participants draw upon their L1 which imposes strict reliance on phonological processing (Fender, 2003). The decrease in Arabic word reading times, particularly at the end of the reading program, showed the use of acquired processing strategies in the target language. The participants' lexical decisions were indicative of the effect of the intervention on Arabic word reading times (Frost, 2005; Kahn-Horwitz et al., 2012; Seymour et al., 2003). Such findings further suggest the flexibility of transferring sub-lexical processing skills in word recognition irrespective of directionality (i.e., L1 to FL; FL to L1).

## 6 Implications and future research

The current experiment has the following pedagogical implication. Adult English language learners may still demonstrate word reading difficulties at university. Therefore, reading interventions at higher-education levels should be designed to customize university students' needs. The findings proffered a new line of research which extends the theoretical framework underlying cross-language transfer of reading skills. For instance, the Cognitive Retroactive Transfer Hypothesis (Abu-Rabia et al., 2013) may integrate online screening tools such as the lexical decision task t measure the potential improvement of cognitive processes deemed important in word recognition. Replications are highly recommended to test the universal and language-specific characteristics of reading fluency. This experiment did not measure the correlation between word recognition and comprehension. To serve this purpose, research must consider typical follow-up tests such as an online incremental sentence processing test (using the moving-window technique). Semantic and syntactic clues (i.e., context effect) may provide more insights into significant role of word recognition in comprehension.

## 7 Conclusion

In summary, the present study measures the effect of a reading training on developing word recognition skills in a bilingual context using linear regression models. The focus of the experiment further investigated the transfer effects of lexical processing strategies from English (FL) to Arabic (L1). We checked for potential betterment in word reading accuracy and speed in Arabic after the intervention The results demonstrate a differential magnitude in the impact the reading intervention exerts on post-accuracy scores in English and Arabic respectively. English word reading accuracy proves to be stronger than Arabic accuracy. This is attributable to print exposure and practice in the target language, rendering accuracy as a script-specific skill. As to reading times, there were palpable variations in word reading speed. Particularly interesting was the adaptability of processing strategies the participants in the treatment group utilize to access items in both languages.

For the experimental group, the transfer was bidirectional, coming from both ways (English to Arabic and vice versa). The participants, by taking more time reading English items, used phonological processing which conformed to the transparency of Arabic orthography. On the contrary, the experimental group was relatively faster in reading Arabic words. This processing strategy is typically associated with processing English words. Such findings additionally expanded the scope recent models of cross-language reading transfer such as the CRT hypothesis, making an innovative contribution in the field. Notwithstanding palpable gains in Arabic word recognition, as demonstrated by the experimental group, post-intervention, these findings should be interpreted with caution. The results reveal the potential for a reverse transfer of processing strategies between the FL (English) and L1 (Arabic), particularly as far as phonological processing is concerned. Nonetheless, given the modest value of improvements in Arabic, also considering the study's sample size, further research is required to measure the robustness and generalizability of such transfer effect.

## 8 Study limitations

It is incumbent upon the experimenters to acknowledge the relatively small sample size (*n* = 45), which renders the generalizability of the findings untenable. While it is true that specialized interventions may well enhance struggling learners' word reading skills, the findings cannot reflect the way readers read in real-life (e.g. read full sentences or stories for comprehension). That is, reading isolated words, using lower-level processes, act as “artificial tasks” and “thus” raise concerns of ecological validity (i.e., bringing the study beyond the scope of the experimental environment). Moreover, there is no guarantee that the acquired skills would be maintained in the long run. This is because the research design (pretest posttest design) utilized in this context does not allow for observing changing patterns at multiple points in time. A cross-sectional or longitudinal design would be more convenient to track readers' progress over time. Finally, the stimuli are simplistic (in their form and shape) and do not measure comprehension *per se*. An incremental word integration task (e.g. a self-paced reading) might have captured the readers' ability to relate word recognition with comprehension.

## Data Availability

The datasets presented in this study can be found in online repositories. The names of the repository/repositories and accession number(s) can be found below: Open Science Framework: https://osf.io/zdh63/.

## Footnotes

^1^This is attributed to the situation of *Diglossia*, which is defined by the co-existence of two spoken varieties in the same community (Ferguson, 1959). Therefore, the experimental stimuli were constructed in MSA since the participants read and write in this language.
