# Supplementary material for: Enhancing word recognition skills in English (FL) and Arabic (L1) through transfer effect: an intervention study
Source: Front Psychol. 2025 Jun 18;16:1564043. doi: 10.3389/fpsyg.2025.1564043 (PMC12213552; doi:10.3389/fpsyg.2025.1564043)
Supplement: Supplementary file 1 [file Data_Sheet_1.pdf]

Supplementary Material

1 SUPPLEMENTARY TABLES AND FIGURES

Table S1. The complete English stimuli.

	Real Words		Pseudowords	
**No.**	**Regular Patterns**	**Irregular Patterns**	**Regular Patterns**	**Irregular Patterns**
1	Reel	Laugh	horble	Thwomb
2	Rack	Friend	Wemp	Quiggle
3	snuggle	Bought	Blorple	jern
4	Bottle	Knee	lavel	Zieve
5	Muzzle	height	Rud	derrial
6	Signal	Bury	Yobble	Sprol
7	Candle	Tough	Plom	lurst
8	bandit	Through	tweg	Choob
9	Shovel	heir	Veffy	Glurn
10	Goggle	Psych	Krilll	Trispe
11	Juggle	Isle	Snard	Strooble
12	Mumble	tomb	Glox	Napsate
13	Wiggle	Colonel	Cliss	Clar
14	Puzzle	Choir	Frabble	Pagbo
15	Napkin	Numb	Zill	Flimberth

Table S2. The complete Arabic stimuli.

Non-vocalized Real words	Vocalized Real words	Non-vocalized Real words	Vocalized Real words
مشعود	أَضْغَبَنِي	ظهر	اِسْتَعَمَ
الظهر	أَرْثَقَتْ	نفذ	أَخْقَفَ
دعوى	أْصْتَمَعَ	قلب	ٱرَتَلَحَتْ
حظيظ	يَتَعَّدُه	اصطدم	مُعْوِصْ
امرا	أُجْتُنْدِبَ	الذلة	تَخَرَّمَ
المقامة	اِنْصَّقَتْ	الرشد	يَنْظَرِبُ
يغل	تَجَهْمَرَ	تولوهم	اِنْطَفَؤَ
كسفا	تَحَرْشَجَ	فيما	اَنْصَرَنْتَ
ألقو	يُكَمْنِل	سنة	لَسَكَا
سخر	دَسَنَتَا	حمل	تَبَشَّمَ
امتثل	غَفْهَعَة	تشكل	ضَمْؤَانٌ
تجمد	نَطَّخَ	انشراح	بَرِظَتْ
تآكل	اِنْصَغْ	تحجج	ثَقَفُّفَاتْ
تمعن	مِشْبَل	تعاهد	غَشْقَمْ
الغداء	مَرْسَجَة	العقار	نَسْتَبْكِ

Table S3. Intervention planning.

Intervention Lessons			
	Units (Examples)		
Consonants	Consonant Phonemes /b/ /d/ /f/ /g/ /h/ /j/ /k/ /l/ /m/ /n/ /p/ /r/ /S/ /t/ /v/ /w/ /y/ /z/	Consonant Blends bl- br- cl- dr- fl- fr- gl- pl- pr- sc- sw- sch- sk- sl- sm- sn- spr- str- ld- lf- lk-	Consonant Diagraphs th- -ng sh- -ch- wh-
Vowels	Long/short Vowels /eɪ/ /iː/ /aɪ/ /juː/ /uː/ /e/ /i/ /ɔ/ /æ/	Diphthongs /ow/ ’cow’ /oy/ ’toy’	R-Controlled Vowels /aː/ car /eɪ/ hare /ɔː/ door
	Sounds and Orthographic Forms ”Vowel Diagraphs”		
Spelling Rules	The sound /k/ ’c’/ ’k’/ ’ic’/ ’qu’/ ’x’/ ’ck’ The sound /əʊ/ ’o – e’/ ’- ow’ ’oa’	Single vowel followers ’-ll’/ ’ss’ ’-dge’/ ’tch’ The sound /aɪ/ ’i -e’ ’-igh’	The sound /eɪ/ ’-ay’ ’a – e’ The sounds /juː/ /uː/ ’u -e’/ ’-ue’ ’-ew’/’-oo’
Syllables	Closed/Open Syllables	Silent e and Vowel Team	R Controlled C+le
	Kettle - Able	The silent e examples include *abuse*, *adore*, and *bore*	Sworn - Wrinkle
	Liver - Baker		Power - Purple
	Metal - Demand	The vowel team includes ow-1 , ea–2 , au-3	Turf - Middle
Word Building	Prefixes	Suffixes	Morphemes
	Frequent prefixes	Roots and Stems	Derivational
	Preposition-based prefixes	Productive Suffixes	Inflectional

Table S4. Sample word reading fluency lesson.

Outline	Learners have to decide if affixes are used correctly while under a time pressure.
Level	Any
Time	10 minutes
Focus	Rapidly recognizing words
Preparation	Prepare a PowerPoint presentation with each slide consisting of one word. You will need 10-20 slides. The words used should be familiar to learners and contain prefixes and/or suffixes (see Notes for variations). Around 20-30% of the words used should be made up and not regularly used in standard English. So, you may use examples such as imbalance, prochoice, prepaid, unchain, and undefeated as ‘correct’ words and examples such as unrecord, unstain, and ungain as made-up words. Programme the presentation so that each slide is only displayed for a second or two. (This can be done through clicking on the ‘transitions’ tab and then using the ‘advance slide after’ function in PowerPoint.)
Procedure	1. Explain to the learners that they will see words on the screen for a very short length of time. Stress that you can no longer control how long the words will be displayed.<br>2. Divide the class into two teams.<br>3. The teams must take turns to shout out Yes (= it is a word) or No (= it isn’t a word) for each item. Anyone in the team can answer.<br>4. Explain that the game will progress very quickly and that there will be no break between items.<br>5. Once you start the PowerPoint presentation try to say nothing and let the learners shout out answers. Answers can be confirmed after the activity.<br>6. Try to keep score and a note of correct and incorrect responses.
Variation 1	Give additional bonus points for answers where a lot of members of one team all shout out the correct answer.
Variation 2	If you wish to avoid competitive games, simply allow the whole class to answer each question.
Variation 3	The same activity can be used without focusing on prefixes and suffixes. For example, at very low levels ‘correct’ words might include and, but, because, and for, while thrit would be an example of an incorrect word.
Variation 4	Essentially the same activity could be used to practice sentence level reading (although an additional second or two would need to be added to the transition time).

Table S5. Sample spelling activity

1	A thief or robber	b_nd_t
2	It could be mathematical or personal	pr_blem
3	A quick meal or snack, with bread	s_ndw_c h
4	Vegetable for Cinderella’s carriage	p_mpk_n
5	One more than twins	tr_pl_ts
6	Open footwear for the summer	s_ndal
7	A brass wind instrument	tr_mp_ t
8	A competition	c_nt_st
9	A serviette	n_pk_n
10	A tooth doctor	d_nt_st
11	Created something original	_nv_nted
12	Like a devil	sat_n_ c
13	Big American river	M_ss_ss_pp_
14	Bad behaviour	m_sc_nd_ct
15	To live in	_nh_b_t

Table S6. The backgound survey.

Please indicate your gender:<br>• Male<br>• Female	Choose your age:<br>• 13-15<br>• 16-17<br>• 21 years older or older
Choose your university level<br>• Semester 2<br>• Semester 4<br>• Semester 6	Choose your English level:<br>• Excellent<br>• Good<br>• Fair<br>• Average<br>• Poor
How long have you been learning English?<br>• 4 years<br>• 5 years<br>• More than 5 years	Do you like reading in English (Ex: books, stories, novels, etc.)?<br>• Yes<br>• No<br>• If your answer is NO, please indicate why?
Choose your Arabic level:<br>• Excellent<br>• Good<br>• Fair<br>• Average<br>• Poor	Do you like reading in Arabic (Ex: books, stories, novels etc.)?<br>• Yes<br>• No
How much time do you usually spend on reading for yourself at home, including books, magazines, newspapers, and materials for work? Check one answer only.<br>• Less than an hour a week<br>• 1-5 hours a week<br>• 6-10 hours a week<br>• More than 10 hours a week	When you are at home, how often do you read for your own enjoyment? Check one answer only.<br>• Every day or almost everyday<br>• Once or twice a week<br>• Once or twice a month<br>• Never or almost never
Which resources do you prefer for reading or completing reading assignments:<br>• Printed (paper) books.<br>• Electronic resources (e-readers, laptops, mobile phones, tablets, etc.)	About how long did it take you to complete this questionnaire? (Write a number. Example 1 minute, 2 minutes, 3 minutes, etc.)
